# Computerized nuclear morphometry in the diagnosis of thyroid lesions with predominant follicular pattern

**DOI:** 10.3332/ecancer.2009.146

**Published:** 2009-09-17

**Authors:** HA Aiad, AG Abdou, MA Bashandy, AN Said, SS Ezz-Elarab, AA Zahran

**Affiliations:** 1 Department of Pathology, Faculty of Medicine, Menoufiya University; 2 Department of Anatomy, Faculty of Medicine, Menoufiya University; 3 Early Cancer Detection Unit, Obstetrics & Gynaecology Hospital, Ain Shams University Hospital; 4 ENT Department, Shebin El-Kom Educational Hospital

**Keywords:** Thyroid, nuclear morphometry, differential diagnosis

## Abstract

**Background:**

Differential diagnosis of thyroid lesions with predominantly follicular pattern is one of the most common problems in thyroid pathology. Development of more objective and reproducible tools for diagnosis is needed. This work is aimed at studying the role of nuclear morphometry in differential diagnosis of different thyroid lesions having predominant follicular pattern.

**Material and methods:**

Semiautomatic image analysis system was used to measure a total of 8 nuclear parameters in 48 thyroid lesions including seven nodular goiter (NG), 14 follicular adenoma (FA), 14 follicular carcinoma (FC) and 13 follicular variant papillary carcinoma (FVPC).

**Results:**

The parameters related to nuclear size (area, perimeter, MaxD, MinD, nuclear size) and shape (L/S ratio, Form_AR) were significantly higher in neoplastic group (FA, FC, FVPC) when compared to non-neoplastic group (NG) P<0.05. The perimeter was the most reliable parameter (area under the cure (AUC)=97%) followed by area, MaxD, and size (all have AUC= 96%) then form-AR (90%), LS ratio (86%) and the least reliable was Min D (79%). Within the neoplastic group, most parameters related to size and shape of the nuclei was significantly higher in FVPC than in FA and FC (p ≤ 0.05). Nuclear area and size (AUC 77%) were the most reliable parameters for differentiation between FVPC and FA. The best cut off values for diagnosing FVPC are nuclear area ≥39.9μm^2^ and nuclear size ≥27.7μm^2^. However, there was no quantitative difference between FC and FA.

**Conclusion:**

Nuclear morphometric parameters may help in the differentiation between neoplastic and non-neoplastic thyroid lesions and between FVPC and follicular neoplasms (FC and FA) but they have no value in the differentiation between FC and FA.

## Background

Differential diagnosis of thyroid lesions with predominantly follicular pattern is one of the most common problems in thyroid pathology [1,2]. Minimally, invasive follicular carcinomas (FC) can be difficult to distinguish from follicular adenomas (FA) by histopathology, since the diagnosis of malignancy depends entirely on the unequivocal demonstration of capsular and/or vascular invasion [3]. Of these, capsular invasion remains a highly controversial issue [4]. Immunohistochemical staining for markers such as galectin-3 [5] and HBME-1 [6] have been suggested as good indicators of thyroid malignancy, although they are not used as absolute markers of malignancy [7]. The follicular variant of papillary thyroid carcinoma (FVPC) is the most common histological subtype of papillary thyroid carcinoma (PC), constituting up to 24% [8]. The histopathological diagnosis of the FVPC is based on the characteristic nuclear features of PC and invasive growth or lymph node metastases [9]. However, one-third of these tumours are totally encapsulated and show no extension beyond the thyroid gland [10]. To complicate the situation further, some FVPC exhibit indicative nuclear features, such as ground glass appearance, only focally, a characteristic common of benign lesions, especially those fixed in high-concentration formalin [11]. Even at the molecular level, FVPC express certain oncogenes in common with follicular tumours [12]. As a result, histopathological diagnosis of the encapsulated FVPC remains one of the most difficult and controversial areas in thyroid surgical pathology [1]. Correct diagnosis in such cases is important since FVPC has the potential for lymphatic and distant metastases [13]. Also, misdiagnosis of an FA as an FVPC will expose the patient to unnecessary aggressive surgical intervention.

Computerized nuclear morphometry is a cost-effective, objective and reproducible tool for evaluation of histological features [14]. Using nuclear morphometry, we can quantify a number of parameters such as those related to nuclear size and shape. The evaluation of these parameters has been claimed to facilitate the diagnosis and management of different neoplasms, including urinary bladder carcinoma [15], skin lymphoma [16], breast carcinoma [17] and soft tissue sarcoma [18]. It has been suggested that nuclear morphometric parameters such as nuclear area and perimeter [19,20], nuclear area coefficient of variation [21] and shape factors [22,23] may allow differentiation between thyroid lesions. Nevertheless, the use of morphometric analysis in thyroid pathology is still very limited in clinical research and does not extend to routine histopathological diagnosis.

## Aim of the study

The aim of this work was to study the role of nuclear morphometry in differential diagnosis of different thyroid lesions having a predominantly follicular pattern.

## Materials and methods

### Case selection

Forty-eight cases of different thyroid lesions were studied retrospectively. Seven cases of nodular goiter (NG), 14 cases of FA, 14 cases of FC (ten of them were minimally invasive) and 13 cases of FVPC (three were the encapsulated variant. We also measured the nuclei of normal thyrocytes from normal thyroid tissue adjacent to hyperplastic nodules from the seven NG cases. All these cases were retrieved from archives of the Pathology Department, Faculty of Medicine, Menoufiya University during the period 1998–2002. We selected the cases on the basis of the presence of a predominant follicular pattern, excluding those with architectural features of classic PC or oncocytic neoplasms. The histological sections were examined by at least two pathologists who agreed the final diagnoses. FVPC and its encapsulated type were diagnosed according to criteria described by Tielens *et al* [9] and Chan *et al* [10]. For follicular carcinoma, unequivocal histological evidence of vascular and/or capsular invasion was documented. Follicular adenoma cases did not show any evidence suggestive of either vascular or capsular invasion regardless of nuclear atypical features. To standardize the process, new 4 μ-paraffin sections were prepared by the same technician, using the same microtome. Morphometric analysis was performed blind, that is without any knowledge of the diagnosis.

### Image analysis system

The image analyser is a semi-automatic system composed of a trinocular microscope (Olympus Corporation, Japan), a digital video camera (Panasonic, Japan) and a personal computer (Toshiba, Japan). The computer is equipped with 3.66 GHz processor with 1022 MB RAM, 160-GB hard disk, NVIDIA Geforce 7600 display adapter, mouse, keyboard, a 17″ high-resolution colour monitor

### System calibration

Morphometric measurements were performed with the help of Digimizer program version 2. Measurements were calibrated in terms of micrometre, using a Nikon micrometre slide before performing any measurements. An image to the slide-stage micrometre (at magnification x1000) was captured and saved on the computer in a JPG file format. The image was used for calibration by opening in Digimizer program window. A straight line measuring 10 μm was copied and pasted to the image for calibration.

### Data collection

**Figure 1:**
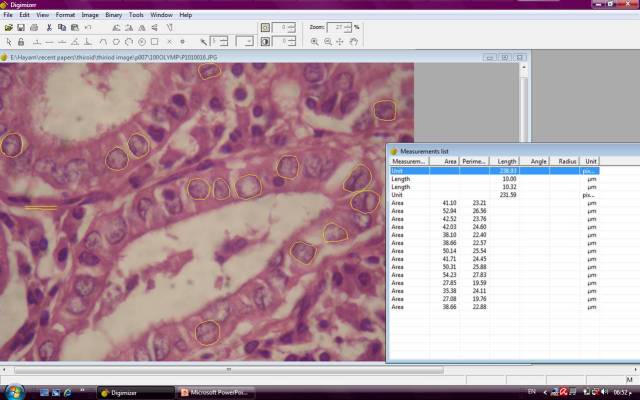
An example of a follicular variant papillary carcinoma image in the Digimizer program window, showing yellow tracing of the nuclei borders and the corresponding area and perimeter values (hematoxylin and eosin x1000, original magnification).

**Figure 2:**
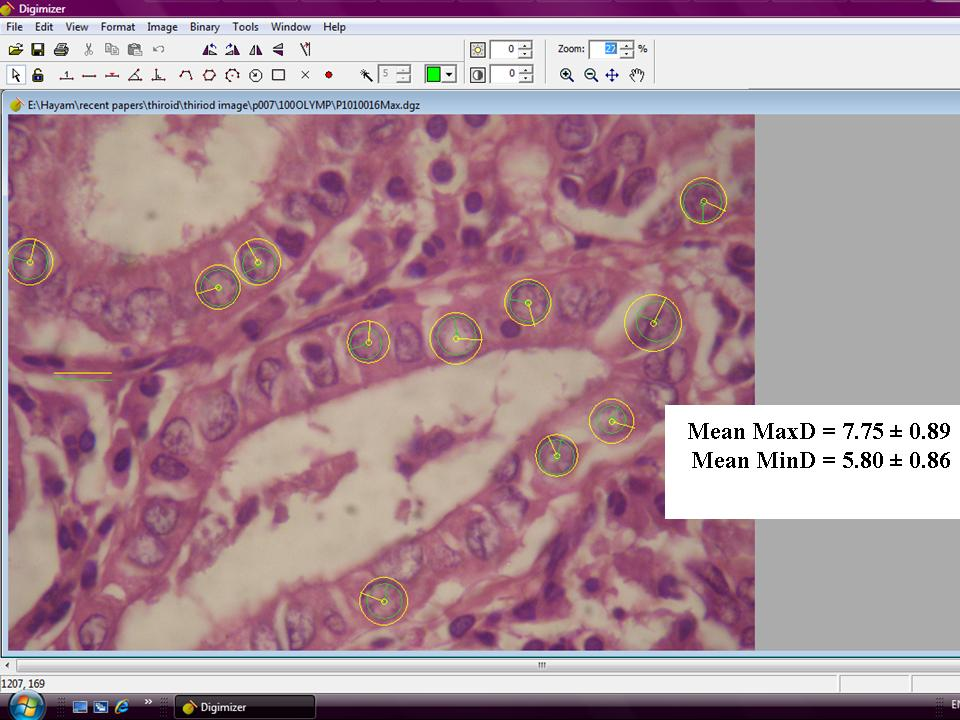
An example of a follicular variant papillary carcinoma image in the Digimizer program window. Diameters of the yellow circles indicate the maximal nuclear diameters (MaxD), while diameters of green circles indicate minimal nuclear diameters (MinD), (hematoxylin and eosin x1000, original magnification).

From the subjectively selected areas, an average of 5–10 microscopic fields, at magnification x1000 were captured for each case. At least 100 nuclei were analysed per slide. Care was taken to include only intact whole nuclei from the actual lesion, avoiding the nuclei of stromal cells. Overlapped and fragmented nuclei were discarded. A total of eight nuclear morphometric parameters were estimated. An actual measurement of four of the nuclear parameters was carried out using the drawing tools in the Digimizer program followed by data extraction and calculation of the remaining four parameters. The measured parameters included: nuclear area (expressed in μm^2^), nuclear perimeter (expressed in μm), maximal nuclear diameter (MaxD) and minimal nuclear diameter (MinD) (Figures 1 and 2]. The calculated parameters included nuclear size (2 x (nuclear area / π)^0.5^) and the coefficient of variation of the nuclear area (NACV) (SD of nuclear area/mean nuclear area x100) expressed as a percentage [21]. The shape factors are calculated by the following formulas; the L/S ratio = MaxD/minD [22] and Form_AR = (1/4) * π * longest axis * shortest axis [17]. In a round circle, the L/S ratio corresponds to one. If the object is elliptic, the L/S ratio is higher than one [22].

### Statistical analysis

The data were coded, entered and processed using the SPSS (version 15) computer program. The level p ≤ 0.05 was considered the cut-off value for significance. Differences between groups were analysed with the unpaired *t* test. We constructed receiver operating characteristic (ROC) curves for the morphometric parameters in order to select cut-off values that best combined sensitivity and specificity for diagnosis of thyroid neoplasia and for diagnosis of FVPC.

## Results

The age of the malignant group (14 FC and 13 FVPC) ranged between 13 and 76 years with a mean ± SD (51.59 ± 14.88). Most of the malignant cases were female with M:F ratio 8:19. The tumour size of malignant cases ranged between 0.3 and 9 cm with a mean ± SD (4.18 ± 2.94). Extra thyroid extension was observed in 10/27 (37%) malignant cases and metastasis was documented in 3/27 (11%). Follicular carcinoma included ten minimally and four widely invasive carcinomas.

Nuclei of normal thyrocytes from normal thyroid tissue adjacent to hyperplastic nodules show area, perimeter, MaxD, MinD, L/S ratio, NACV, Nuclear size and Form_AR as follow: 19.43 ± 2.76, 15.15 ± 1.13, 4.49 ± 0.18, 4.21 ± 0.55, 1.12 ± 0.15, 21.11 ± 4.93, 4.97 ± 0.34, 14.85 ± 1.92, respectively. These values were not significantly different from those of nuclei of NG cells (p > 0.05).

The neoplastic group (FA, FC, FVPC) showed significantly higher mean values of nuclear parameters related to size (area, perimeter, MaxD, MinD, nuclear size) and shape (L/S ratio, Form_AR) when compared to the non-neoplastic group (nodular goiter) p < 0.05 (Table 1).

Within the neoplastic group, parameters related to size (area, perimeter, MaxD, nuclear size) and shape of the nuclei (Form_AR) were significantly higher in FVPC than in FA and FC (p ≤ 0.05) (Table 2). However, there was no significant difference between FA and FC as regards parameters related to either size or shape (**p** > 0.05).

**Figure 3:**
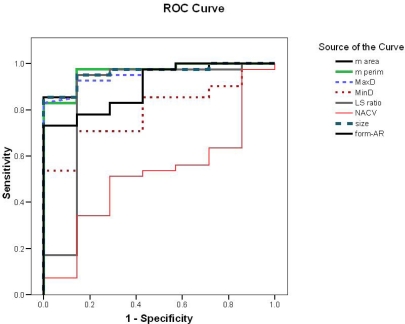
Receiver operating characteristic curve for differentiation of neoplastic from non-neoplastic thyroid lesions.

According to the ROC curve (Figure 3, Table 3), the perimeter was the most reliable parameter for differentiating neoplastic from non-neoplastic lesions (area under the curve (AUC) = 97%) (95% CI 91–100%) followed by area, MaxD and size (all have AUC = 96%) (95% CI 91–100%, 90–100% and 91–100%). Form-AR (90%), LS ratio (86%) (95% CI 65–100%) and last of all MinD (79%) (95% CI 65–94%) p < 0.05 were the least reliable. The NACV was not reliable for diagnosing thyroid neoplasia (AUC = 52%) (95% CI 30–74%) p > 0.05. The best cut-off values for the reliable parameters and their diagnostic validity are listed in Table 3.

**Figure 4:**
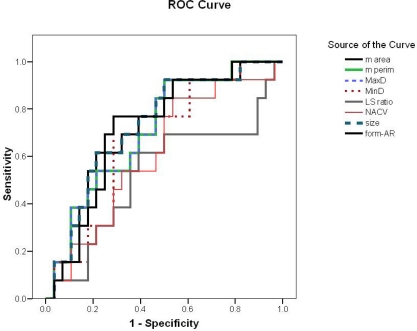
Receiver operating characteristic curve for differentiation of FVPC from FA

According to the ROC curve for differentiation of FVPC from FA, nuclear area and size were the most reliable parameters (AUC = 77%) (Figure 4, Table 4). The best cut-off values for diagnosing FVPC are nuclear area ≥ 39.9 μm^2^ and nuclear size ≥ 27.7 μm^2^. The diagnostic validity of these values is shown in Table 5. For differentiation of FVPC from FC, all parameters were fair as the AUC for all were less than 75% and no cut-off value was selected.

## Discussion

The subjective evaluation of cytological atypical features is not a reliable criteria for malignancy in thyroid lesions since these changes may be present in benign lesions such as adenomatous hyperplasia and follicular adenoma [1]. Therefore, it is important to find an objective morphological analysis to allow differential diagnosis between benign and malignant lesions in the thyroid gland. In this study, we estimated different nuclear morphometric parameters in thyroid lesions that pose diagnostic problems in thyroid pathology. We selected lesions showing follicular pattern excluding those with architectural features of classic PC. Oncocytic neoplasms were also excluded because these constitute distinct entities [23]. In our nuclear morphometric analysis, parameters related to both size and shape of the nuclei were significantly higher in neoplastic than non-neoplastic groups (nodular goiter). An ROC curve confirmed the reliability of most of the studied nuclear parameters in predicting the neoplastic nature of the lesion. Our findings agree with previous studies supporting the validity of morphometry in diagnosis of thyroid neoplasia [24,25]. More importantly, we could statistically select cut-off values suitable for diagnosing thyroid neoplasia with increased sensitivity and specificity. More studies are recommended to test the validity of such cut-off values in histopathological and cytological preparations to prove its diagnostic utility.

In the current study, the nuclei of FVPC were significantly larger than those of follicular neoplasms (FA and FC). Similarly, previous morphometric studies succeeded in discrimination of papillary carcinoma in general or its follicular variant in particular from follicular neoplasms [23, 24, 26]. The differentiation between encapsulated FVPC and FA is one of the documented problems in thyroid pathology [1–3]. The completely encapsulated FVPC still carry the potential of lymph node and distant metastasis [13]. The term ‘well-differentiated tumour of uncertain malignant potential’ has been recommended for encapsulated follicular tumours with suggestive nuclear features of PC [27]. Many immunohistochemical stains were suggested as markers of PC. Cytokeratin 19 is one of the promising markers, but it is also positive in significant number of benign lesions and at sites of previous biopsy [28,29]. Immunostaining for RET to identify RET/PTC rearrangement was also reported, but it still is dependant on availability of reliable and sensitive antibodies [30]. In the current study, the nuclei of FVPC were significantly larger and more irregular in shape than those of FA and the ROC curve demonstrated the reliability of nuclear area and size in diagnosing FVPC. Moreover, we suggested cut-off values ≥ 39.9 μm^2^ and ≥ 27.7 μm^3^ for nuclear area and size, respectively, with best sensitivity and specificity. Our findings demonstrate that quantitative measurements of nuclear parameters may disclose more features than that can be detected by the pathologistís subjective examination. Applying such cut-off values could be a more reproducible and objective method compared to the subjective detection of papillary nuclear features. It is probably more cost-effective than immunohistochemistry and may avoid some of the errors associated with this technique.

The FC can be difficult to distinguish from FVPC. In a number of follicular tumours with definite capsular and vascular invasion, the nuclei may show features suggestive of PC. The diagnosis of ‘well-differentiated carcinoma not otherwise specified’ was proposed for those tumours [27]. Reviewing the literature, some studies did not find a difference between nuclear parameters in papillary and follicular carcinomas [26, 31]. In contrast, Karslioğlu *et al* found that most of the parameters related to nuclear size, but not nuclear shape was significantly higher in PC than FC in cytological samples [23]. In our study, nuclei of FVPC did show significant difference from those of FC; however, we could not select suitable cut-off values as all the parameters showed AUC less than 75%. Although the clinical behaviour of FVPC is better than FC [8], the distinction between both tumours may be clinically irrelevant since the treatment is similar and depends upon the extent of invasion [32].

We showed that quantitative nuclear assessment did not help much to solve the problem of differentiating FC from FA. Similarly, some studies concluded that there was considerable overlap of nuclear morphometric parameters between FC and FA [25,33]. Gupta *et al* studied different nuclear morphometric parameters in thyroid lesions and showed highly significant differences between benign and malignant groups; however, the lowest sensitivity and specificity in their study was documented between FC and FA [24]. In contrast, others had found significant differences between both tumours using quantitative analysis [23, 31, 34, 35]. In our study, the inclusion of FA cases showing some atypical nuclear features may be responsible for the absence of significant differences between FA and FC, while exclusion of these cases from the previous studies may affect their findings [23,31].

In the present study, we used a semi-automatic system that could be easily constructed in any pathology laboratory. The morphometric measurements for each case took approximately 30–45 min. There is no doubt that automated image analysers could perform these measurements much quicker, but due to their cost we resorted to the partially automated approach. In addition, even with the automatic image analysers, we need to manually trace the nuclei to avoid the complexity of histological images. For example, some nuclei may not be easily delineated if their edges blend into the light density of the cytoplasm [36].

## Conclusion

Our results draw the attention to the use of nuclear morphometry in the diagnosis of difficult follicular-patterned lesions of the thyroid gland. Nuclei of neoplastic group showed significantly higher values than those of non-neoplastic group. Although nuclei of FVPC showed significant quantitative difference in size and shape from those of FC and FA, we could achieve sharp cut-off values between FVPC and FA only. The most reliable parameters are the nuclear area and size. Lesions with a mean nuclear area ≥ 39.9 μm^2^ and a mean nuclear size ≥ 27.7 μm^2^ are most probably papillary carcinoma rather than FA. We agree that our figures cannot serve as absolute diagnostic criteria since they are only based on statistical differences. However, further application on larger studies to find suitable cut-off numerical values would open the gate for application of quantitative evaluation in the routine diagnostic pathology of thyroid lesions.

## Figures and Tables

**Table 1:**
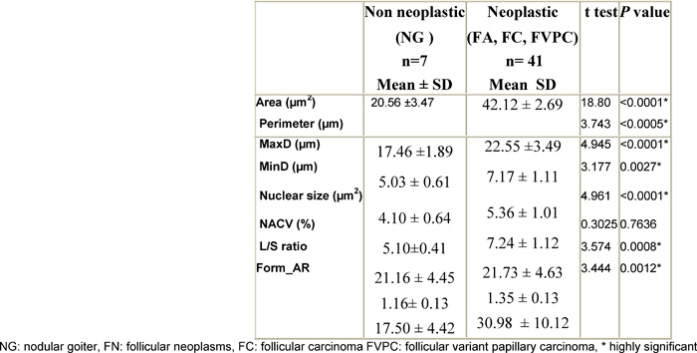
Comparison of nuclear morphometric parameters between non-neoplastic and neoplastic groups

**Table 2:**
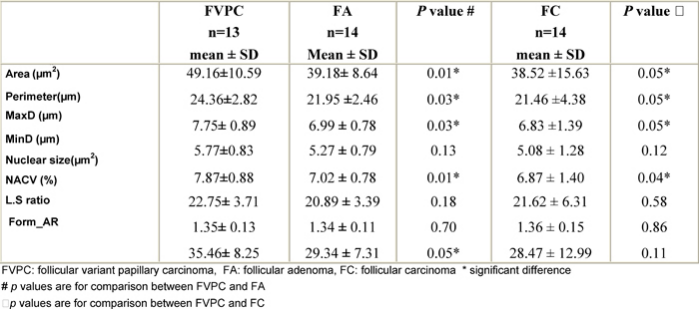
Comparison of nuclear morphometric parameters within the neoplastic group

**Table 3:**
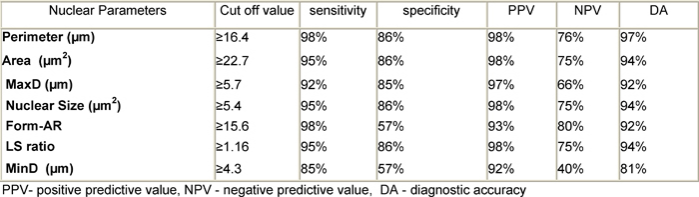
The best cut-off values selected for diagnosis of thyroid neoplasia

**Table 4:**
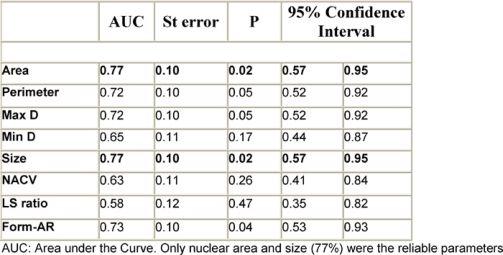
The diagnostic reliability of different nuclear parameters in differentiation of FVPC from FA

**Table 5:**
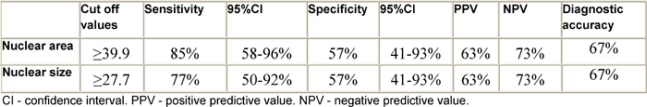
Cut-off values and sensitivity and specificity of nuclear area and size in differentiation of FVPC from FA
